# Targeting the NLRP3 inflammasome to attenuate spinal cord injury in mice

**DOI:** 10.1186/s12974-017-0980-9

**Published:** 2017-10-25

**Authors:** Wu Jiang, Maoqiang Li, Fan He, Shaobo Zhou, Liulong Zhu

**Affiliations:** Hangzhou First People’s Hospital, Nanjing Medical University, No. 261 Huansha Road, Shangcheng District, Hangzhou, 310006 China

**Keywords:** NLRP3 inflammasome, Inflammation, Spinal cord injury, BAY 11-7082, A438079

## Abstract

**Background:**

Spinal cord injury (SCI) is a devastating disease, which results in tissue loss and neurologic dysfunction. NLRP3 inflammasome plays an important role in the mechanism of diverse diseases. However, no studies have demonstrated the role of NLRP3 inflammasome and the effects of NLRP3 inflammasome inhibitors in a mouse model of SCI. We investigated whether inhibition of NLRP3 inflammasome activation by the pharmacologic inhibitor BAY 11-7082 or A438079 could exert neuroprotective effects in a mouse model of SCI.

**Methods:**

SCI was performed using an aneurysm clip with a closing force of 30 g at the level of the T6-T7 vertebra for 1 min. Motor recovery was evaluated by an open-field test. Neuronal death was assessed by terminal deoxynucleotidyl transferase dUTP nick end labeling and Nissl staining. Mitochondrial dysfunction was determined by quantitative real-time polymerase chain reaction (qPCR), western blot, and detection of mitochondrial membrane potential level. Microglia/macrophage activation and astrocytic response were evaluated by immunofluorescence labeling.

**Results:**

Inhibition of NLRP3 inflammasome activation by pharmacologic inhibitor BAY 11-7082 or A438079 reduced neuronal death, attenuated spinal cord anatomic damage, and promoted motor recovery. Furthermore, BAY 11-7082 or A438079 directly attenuated the levels of NLRP3 inflammasome and proinflammatory cytokines. Moreover, BAY 11-7082 or A438079 alleviated microglia/macrophage activation, neutrophils infiltration, and reactive gliosis, as well as mitochondrial dysfunction.

**Conclusions:**

Collectively, our results demonstrate that pharmacologic suppression of NLRP3 inflammasome activation controls neuroinflammation, attenuates mitochondrial dysfunction, alleviates the severity of spinal cord damage, and improves neurological recovery after SCI. These data strongly indicate that the NLRP3 inflammasome is a vital contributor to the secondary damage of SCI in mice.

## Background

Traumatic spinal cord injury (SCI) is a devastating disease that results in deficits in human body functions [1]. Unfortunately, there is no effective treatment for SCI [2, 3]. Accumulating evidence suggests that secondary damage of SCI is orchestrated by various pathophysiologic mechanisms, including inflammation [4], mitochondrial dysfunction [5], and oxidative stress [6]. However, the detailed molecular mechanisms that underlie SCI are not completely understood.

The NOD-like receptor protein-3 (NLRP3) inflammasome, which is assembled by NLRP3, apoptosis-associated speck-like protein containing a caspase recruitment domain (ASC), caspase-1 after an endogenous “danger signal,” and exogenous infection, is an important cytosolic protein complex [7] which provides a caspase-1-activation platform to promote the maturation and release of interleukin (IL)-1β and IL-18 [8]. NLRP3 inflammasome plays a substantial role in the neuroinflammation of Alzheimer’s disease in mice [9]. Moreover, NLRP3 inflammasome activation contributes to mitochondrial dysfunction in a mouse model of albumin-induced renal tubular injury [10].Additionally, De Rivero and coworkers have reported that NOD-like receptor protein-1 (NLRP1) inflammasome is activated post SCI and that the neutralization of ASC led to a significant suppression in the activation of caspase-1 and processing of IL-1β and IL-18 [11].We and others have recently shown that SCI triggers NLRP3 inflammasome activation in spinal cord tissue [12, 13] and spinal cord microglia [14]. The mRNA and protein expression of NLRP3 inflammasome are significantly rose 3 days post injury in a rat model after SCI [12, 13]. Moreover, NLRP3 and ASC are expressed in neurons, microglia, and astroglia, and neurons and microglia are major sources of the NLRP3 and ASC, respectively [13]. d-β-Hydroxybutyrate relieved pain hypersensitivity and promoted functional recovery in mice with SCI, possibly through inhibition of NLRP3 inflammasome activation [15]. Wogonoside attenuates SCI-induced neuroinflammation by inhibiting NF-κB and NLRP3 inflammasome activation [16]. No studies have demonstrated the role of NLRP3 inflammasome and the effects of NLRP3 inflammasome inhibitors in a mouse model of SCI. Hence, the present study aimed to test whether inhibition of NLRP3 inflammasome activation by pharmacologic inhibitor BAY 11-7082 or A438079 could exert neuroprotective effects after SCI in mice.

## Methods

### Animals

Animal experiments were conducted in female C57BL/6 mice (25–30 g, 8–10 weeks) based on previous study [4]. All animal procedures were approved by the Ethics Committee of Hangzhou First People’s Hospital for Animal Experiment. Animals were maintained under controlled conditions with a 12-h light/dark cycle at 23 °C with access to food and water ad libitum.

### Surgical preparations

Mice were anesthetized with ketamine (75 mg/kg) and xylazine (3 mg/kg) intraperitoneally and the muscles overlying the T5 to T8 vertebrae were dissected, followed by laminectomy to expose the spinal cord. To induce SCI, extradural compression of the spinal cord at the level of the T6-T7 vertebra was conducted via placement of an aneurysm clip with a closing force for 1 min, as described by Paterniti et al. [17]. The aneurysm clip is with a closing force of 30 g based on previous study [18]. Mice undergoing laminectomy alone served as the sham group.

Mice in treatment groups received a daily single-dose intraperitoneal injection of BAY 11-7082 (Tocris Bioscience, Bristol, UK) at 20 mg/kg or A438079 (Tocris Bioscience) at 80 mg/kg in 0.1 ml vehicle (DMSO and 0.9% NaCl, 1:3) immediately after injury and following 2 days. Sham and SCI groups received daily 0.1 ml vehicle immediately post injury and following 2 days intraperitoneally. Mice underwent manual bladder expression twice a day until recovery of reflex bladder emptying. The timing and dose of BAY 11-7082 [19, 20] and A438079 [21] were based on previous studies.

### Behavioral assessments

Hind limb locomotor function in an open field was assessed with the Basso Mouse Scale (BMS) [22], using a 0- to 9-point scale (complete paralysis to normal hind limb function), by two experienced investigators who were blinded to experimental treatment and observed open-field locomotion for over 4 min on days 1, 3, and 7 post injury, then once weekly thereafter for next 5 weeks (*n* = 9 mice/group).

### Paraffin section preparation

Spinal cord samples (1 cm with injury epicenter located centrally) were dissected out after transcardial perfusion, fixed in paraformaldehyde solution at room temperature, dehydrated, and embedded in paraffin 3 days after SCI. Four micrometer-thick transverse paraffin sections (proximity to and located in the injury site) were used for Nissl staining, immunofluorescence labeling, and terminal dexynucleotidyl transferase-mediated dUTP nick end labeling (TUNEL).

### Nissl staining

For Nissl staining, 4-μm-thick paraffin sections 72 h post-SCI were underwent deparaffinization, stained with cresyl violet, dehydrated with different concentrations of ethanol, cleared in xylene, and mounted with neutral balsam. Cell counts in the ventral horn at the epicenter of the lesion and 0.5, 1, 1.5, and 2 mm rostral and caudal to the injury epicenter were calculated and analysis was through ImageJ software (1.4, NIH). The number of positive cells from above field (high-power field, 450 um × 325 um) was averaged (*n* = 5 mice/group).

### Immunofluorescence labeling

After deparaffinization, sections were performed with antigen retrieval in 0.01 M citrate buffer solution (pH = 6.0), followed by incubation overnight with primary antibodies anti-Iba1 (1:500, Abcam, Cambridge, UK) and anti-glial fibrillary acidic protein (GFAP) (1:500, Abcam) and then incubated with secondary antibodies. Images were obtained with the fluorescence microscope (Olympus, Tokyo, Japan). Cell counts and analysis were through ImageJ software (1.4, NIH). The number of positive cells from four optical fields (high-power field, 225 um × 162 um) in each of the four sections per animal were averaged (*n* = 5 mice/group).

### TUNEL

TUNEL staining was performed using an In Situ Cell Death Detection kit (Roche, Basel, Switzerland) according to the manufacturer’s instructions. Briefly, after deparaffinization, slides were incubated with proteinase-K, then TUNEL reaction mixture, followed by blocking buffer with peroxidase-streptavidin conjugate solution, and finally 0.03% diaminobenzidine. For co-staining of surfactant proteins, NeuN and TUNEL, the slices were first labeled with the anti-NeuN (1:500, Abcam), and then TUNEL, subsequently underwent nuclear staining with DAPI. Images were examined by a fluorescence microscope (Olympus, Tokyo, Japan). Cell counts and analysis were through ImageJ software (1.4, NIH). The number of positive cells from four optical fields (high-power field, 450 um × 325 um) in each of the four sections per animal were averaged (*n* = 5 mice/group).

### Extraction of fresh tissue and mitochondria preparation

Spinal cord tissue (1 cm with injury epicenter located centrally) was carefully removed and stored at − 80 °C until use for quantitative real-time PCR (qPCR), western blot, and myeloperoxidase (MPO) activity detection.

The spinal cord tissue was harvested and homogenized with isolation buffer (Beyotime Institute of Biotechnology, Shanghai, China) in ice-chilled Dounce homogenizers (1:10, *w*/*v*), and then centrifuged at 1000*g* for 5 min. Supernatants were transferred into another tube, centrifuged at 8000*g* for 10 min, subsequently removed and centrifuged at 12000*g* to acquire cytosol fractions. Mitochondria-enriched pellets were resuspended and washed with isolation buffer, subsequently re-pelleted by centrifugation at 1000*g* for 5 min and 8000*g* for 10 min. The cytosol fraction was used for determining cytosolic cytochrome c (Cyt C) levels.

### Detection of mitochondrial membrane potential

Mitochondrial membrane potential (MMP) level was assessed using JC-1 MMP detection kit (Genmed Scientifics Inc., Shanghai, China) by detection of fluorescence intensity with a fluorescence spectrophotometer (exCitation 490 nm, emission 520 nm) based on previous study [23].

### Quantitative real-time PCR

Total RNA and DNA was isolated using Trizol reagent (Invitrogen, Carlsbad, CA, USA) and a DNeasy Tissue Kit (Qiagen, Valencia, CA, USA), respectively. All primers used were designed through Primer 3 software (Table 1). The mRNA expression levels of genes and mitochondrial (mt) DNA copy number was detected. For qPCR of mRNA expression levels of genes and mt DNA copy number, reverse transcription was carried out, followed by real-time PCR amplification. The copy number of mtDNA expression and ATP synthase mRNA expression were normalized against the 18S rRNA (encoded by nuclear DNA) level; other mRNA expression levels were normalized against reference gene GAPDH and measured using the ∆∆CT method (*n* = 5 mice/group).

**Table 1:** Real-time PCR primer sequences

Gene	Forward primer (5′–3′)	Reverse primer (5′–3′)
NLRP3	CTCCAACCATTCTCTGACCAG	ACAGATTGAAGTAAGGCCGG
ATP synthase	TCCATCAAAAACATCCAGAAAA	GAGGAGTGAATAGCACCACAAA
Mitochondrial DNA	TTTTATCTGCATCTGAGTTTAATCCTGT	CCACTTCATCTTACCATTTATTATCGC
18S	TTCGGAACTGAGGCCATGATT	TTTCGCTCTGGTCCGTCTTG
iNOS	CGCTTGGGTCTTGTTCACT	TCTTTCAGGTCACTTTGGTA
Arginase1	GCTTGCTTCGGAACTCAAC	CGCATTCACAGTCACTTAGG
GAPDH	TCATGGATGACCTTGGCCAG	GTCTTCACTACCATGGAGAAGG

### MPO activity detection

MPO, a representative marker for the activation and infiltration of neutrophils, was measured using a commercial assay kit according to the manufacturer’s protocol (Jiancheng Co., Nanjing, China) in the spinal cord tissue (*n* = 5 mice/group).

### ELISA

Spinal cord samples (*n* = 5 mice/group) were homogenized in phosphate-buffered saline (PBS), subsequently centrifuged at 5000×*g* at 4 °C for 10 min. IL-1β, IL-18, and tumor necrosis factor (TNF)-α concentrations in the supernatant were detected using enzyme-linked immunosorbent assay (ELISA) kits (R&D Systems, Minneapolis, MN, USA).

### Western blot

For protein sample preparation, spinal cord specimens were homogenized and extracted with RIPA buffer (Beyotime, Nanjing, Jiangsu, China). Protein concentration was measured with a BCA™ protein assay kit (Pierce, Bonn, Germany) according to the manufacturer’s instructions. Total protein (30 μg/lane) was separated through sodium dodecyl sulfate polyacrylamide gel electrophoresis, and then transferred to polyvinylidene difluoride membranes (Millipore, Bedford, MA, USA). Thereafter, membranes were blocked with 5% skimmed milk, and then incubated with the following primary antibodies: anti-NLRP3, anti-ASC, anti-caspase-1 (all 1:1000; Santa Cruz Biotechnology, Santa Cruz, CA, USA), and β-actin (1:1000; Santa Cruz Biotechnology) overnight at 4 °C, followed by incubation with the respective secondary antibody. Moreover, the level of cytosolic Cyt C (1:1000; Abcam, Cambridge, UK) was also determined. The bands were visualized using an ECL kit (Millipore, Bedford, MA). For densitometric quantification, the specific band intensities were normalized to β-actin in the same blot (*n* = 5 mice/group).

### Statistical analysis

All values are presented as the mean ± standard error of the mean (SEM). Statistical differences between groups were analyzed using one-way analysis of variance (ANOVA) followed by Tukey’s post hoc test. When the values in the study were not normally distributed, statistical differences were analyzed using one-way ANOVA on ranks with post hoc Dunn’s method. BMS score was analyzed using two-way ANOVA with Bonferroni post hoc tests. A *p* value of less than 0.05 was considered statistically significant.

## Results

### Time course of NLRP3 inflammasome after SCI

**Fig. 1:**
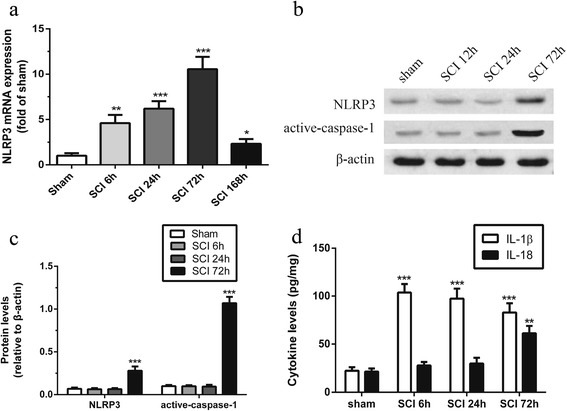
Time course of NLRP3 expression in spinal cord tissue after SCI. **a** Time course of NLRP3 expression during 7 days post-SCI analyzed by qPCR. **b**, **c** Representative western blots and quantification analysis of NLRP3 and active-caspase-1 during 3 days post-SCI. **d** Time course of interleukin (IL)-1β and IL-18 levels during 7 days post-SCI analyzed by ELISA. *p < 0.05, **p < 0.01, ***p < 0.001. Data represent means ± SEM, *n* = 5

To determine the profile of NLRP3 inflammasome, we analyzed the mRNA of NLRP3 6, 24, 72, and 168 h post-SCI and protein expression of NLRP3 6, 24, and 72 h post-SCI (Fig. 1a–c). NLRP3 mRNA level immediately rose within the first 6 h (*P* < 0.01) and reached its peak value 3 days post injury compared to sham controls (Fig. 1a, *P* < 0.001), and NLRP3 and active-caspase-1 protein level were significantly elevated 3 days after SCI (Fig. 1b, c, *P* < 0.01). In addition, the pro-caspase-1 protein expression did not significantly change in every group (data not shown). The protein levels of IL-1β and IL-18 were analyzed using ELISA based on previous studies [12, 13]. IL-1β concentration strongly rose at 6 h (Fig. 1d, *P* < 0.001) and slightly declined but were still significantly increased at 24 h (Fig. 1d, *P* < 0.001) and 3 days (Fig. 1d, *P* < 0.001) post injury compared to sham group. IL-18 concentration is significantly increased at 3 days after SCI (Fig. 1d, *P* < 0.001).

### Effects of NLRP3 inflammasome blockage on behavioral recovery

**Fig. 2:**
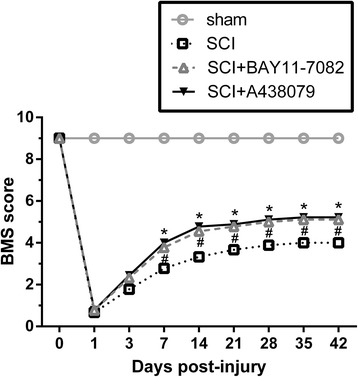
BAY 11-7082 or A438079 improves locomotor functional recovery. Basso Mouse Scale (BMS) score after spinal cord injury (SCI). ^#^ *p* < 0.05, SCI+ BAY 11-7082 vs. SCI. *p < 0.05, SCI+ A438079 vs. SCI. Data are presented as the mean ± SEM, *n* = 9

Behavioral recovery was analyzed using BMS score (Fig. 2). SCI mice had lower scores than the sham mice (Fig. 2, *P* < 0.001), indicating that SCI mice were significantly impaired 1 day post injury. Treatment with BAY 11-7082 or A438079 significantly improved locomotor functional recovery with increasing the BMS score 7 days after injury (Fig. 2, *P* < 0.05); this difference was maintained up to 6 weeks after injury (Fig. 2, *P* < 0.05).

### Effects of NLRP3 inflammasome blockage on neuron death

**Fig. 3:**
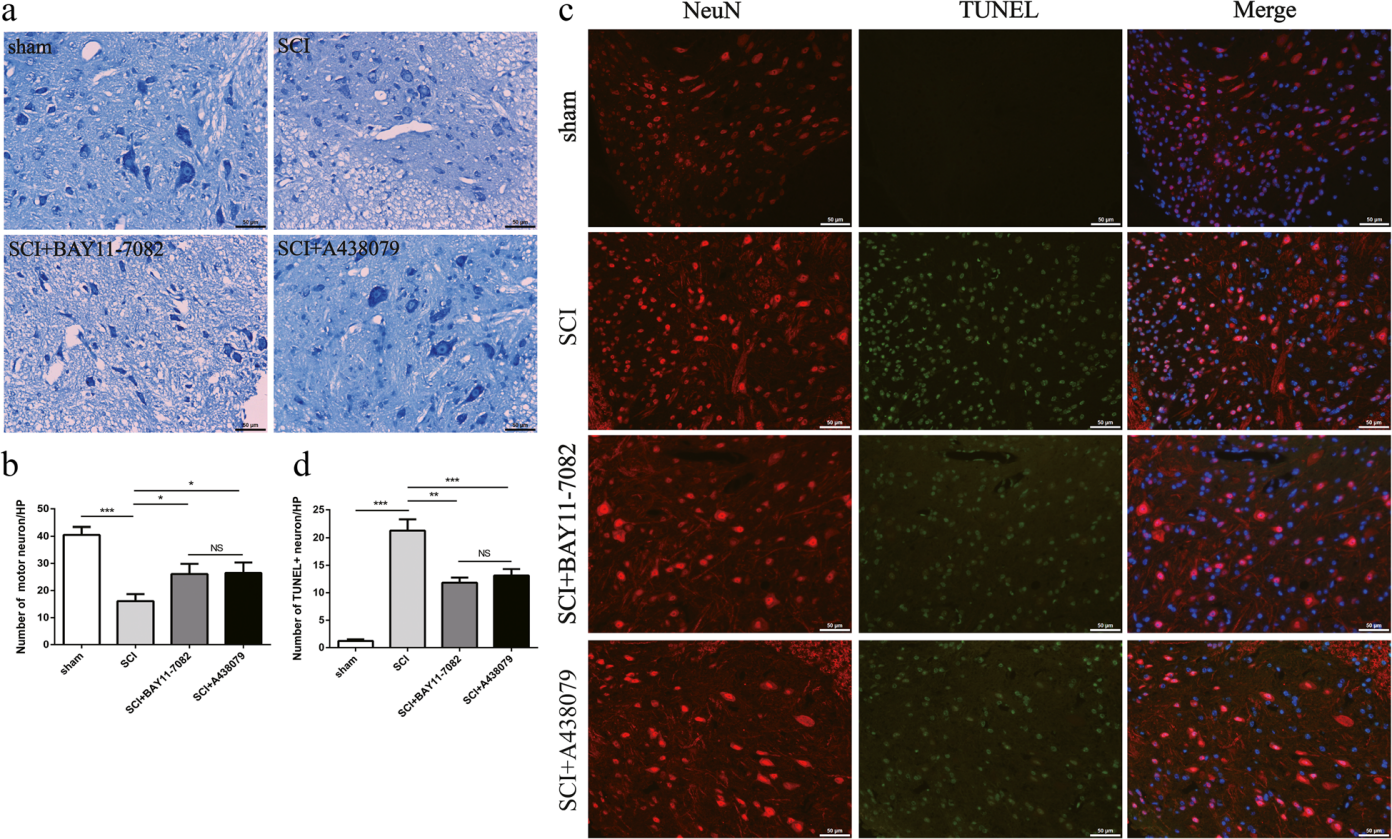
BAY 11-7082 or A438079 attenuates neuronal death. **a** Nissl staining to estimate the loss of neurons 3 days post injury, representative images 2 mm rostral to the lesion epicenter. **b** Quantitative estimation of ventral motor neuron (VMN). **c** TUNEL (green fluorescence)/NeuN (red fluorescence)-positive cells in the ventral horn 3 days post injury. **d** Quantitative estimation of TUNEL-positive neurons. HP (high-power field, 450 um × 325 um). *p < 0.05, **p < 0.01, ***p < 0.001, NS *p* > 0.05. Data are presented as the mean ± SEM, *n* = 5. Scale bars are 50 μm

To determine the effects on survival of motor neurons, Nissl staining and co-staining with NeuN (red fluorescence) and TUNEL (green fluorescence) were performed 3 days post SCI. As shown in Fig. 3a, the number of Nissl-positive cells in the ventral horn was obviously decreased in the SCI group (*P* < 0.001). However, BAY 11-7082 (Fig. 3a, b, *P* < 0.05) or A438079 (Fig. 3a, b, *P* < 0.05) significantly attenuated this effect. The number of TUNEL-positive neurons in the ventral horn area was evidently increased following SCI (Fig. 3c, d, *P* < 0.001), whereas BAY 11-7082 (*P* < 0.01) or A438079 (*P* < 0.001) clearly decreased this effect (Fig. 3c, d). Furthermore, there was no significant difference between BAY 11-7082 and A438079 treatment (*P* > 0.05).

### Effects of NLRP3 inflammasome blockage on microglia/macrophage

**Fig. 4:**
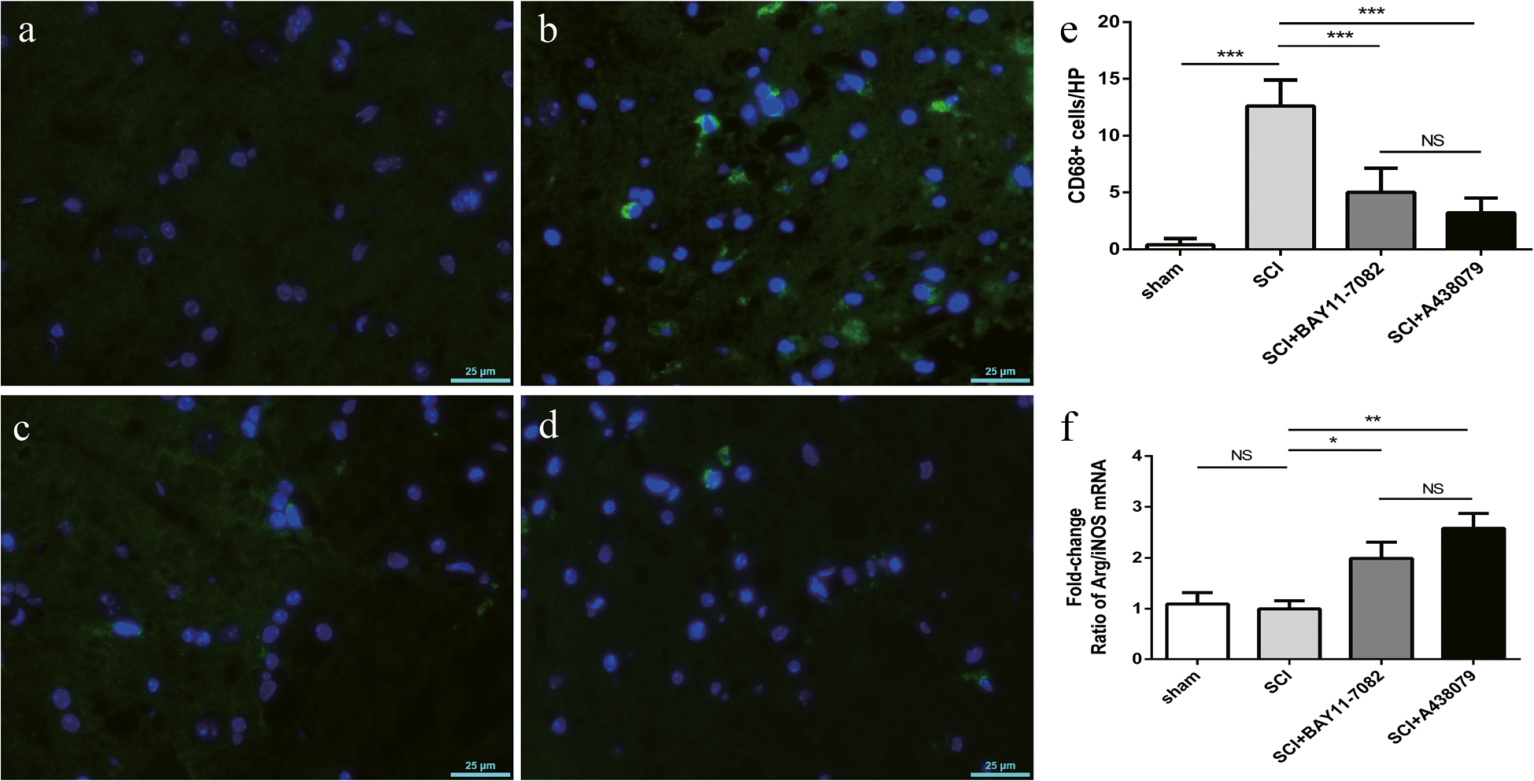
BAY 11-7082 or A438079 attenuates microglia/macrophage activation and may regulate microglia/macrophage polarization 3 days after SCI. (**a**-**d**) Immunofluorescence labeling to estimate the number of CD68-positive cells(green fluorescence) 3 days after SCI. a:SCI;b:sham;c:SCI+ BAY11-7082; d:SCI+ A438079. (**e**) Quantification analysis of CD68-positive cells. (**f**) Quantification analysis of the ratio of Arg1/iNOS mRNA. HP (High power field, 225um×162um). *p < 0.05, **p < 0.01, ***p < 0.001, NS *p* > 0.05. Data are presented as the mean ± SEM, *n*=5. Scale bars are 25 μm

To determine the number of activated microglia/macrophage, CD68 expression was estimated by immunofluorescence labeling (Fig. 4a–d, green fluorescence) 3 days post SCI [2, 24]. SCI induced a significant increase in the number of activated microglia/macrophage, as demonstrated by an elevation in CD68-positive cells (Fig. 4b, e, *P* < 0.001). Such an alteration was remarkably reversed by BAY 11-7082 (Fig. 4c, e, *P* < 0.01) or A438079 (Fig. 4d, e, *P* < 0.01).

Based on previous study [16], we performed qPCR of markers of the M1 phenotype microglia/macrophage (inducible nitric oxide synthase, iNOS) [4, 13, 25] or M2 phenotype microglia/macrophage (arginase1, Arg1) [4, 13, 25] and calculated the ratio of Arg1/iNOS mRNA to analyze the change of microglia/macrophage polarization 3 days post injury. We found that BAY 11-7082 (*P* < 0.05) or A438079 (*P* < 0.01) increased the ratio of Arg1/iNOS mRNA (Fig. 4f). Furthermore, there was no significant difference between SCI and sham groups, and BAY 11-7082 and A438079 treatment (*P* > 0.05).

### Effects of NLRP3 inflammasome blockage on neutrophils infiltration

**Fig. 5:**
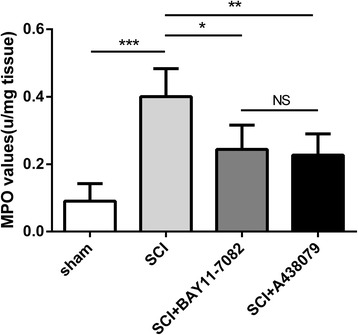
BAY 11-7082 or A438079 inhibits neutrophils infiltration 3 days after SCI. Quantification analysis of MPO activity. *p < 0.05, **p < 0.01, ***p < 0.001, NS *p* > 0.05. Data are presented as the mean ± SEM, *n* = 5

Myeloperoxidase (MPO), which is involved in the catalysis and formation of ROS [26], represents the activation and infiltration of neutrophils [27]. Based on previous study [28], we investigated MPO activity to analyze neutrophil infiltration in this study. SCI induced a significant increase of MPO activity (Fig. 5, *P* < 0.001). However, such alteration was remarkably reversed by BAY 11-7082 (Fig. 5, *P* < 0.05) and A438079 treatment (Fig. 5, *P* < 0.01). Moreover, no significant difference was found between BAY 11-7082 and A438079 treatment (*P* > 0.05).

### Effects of NLRP3 inflammasome blockage on reactive gliosis

**Fig. 6:**
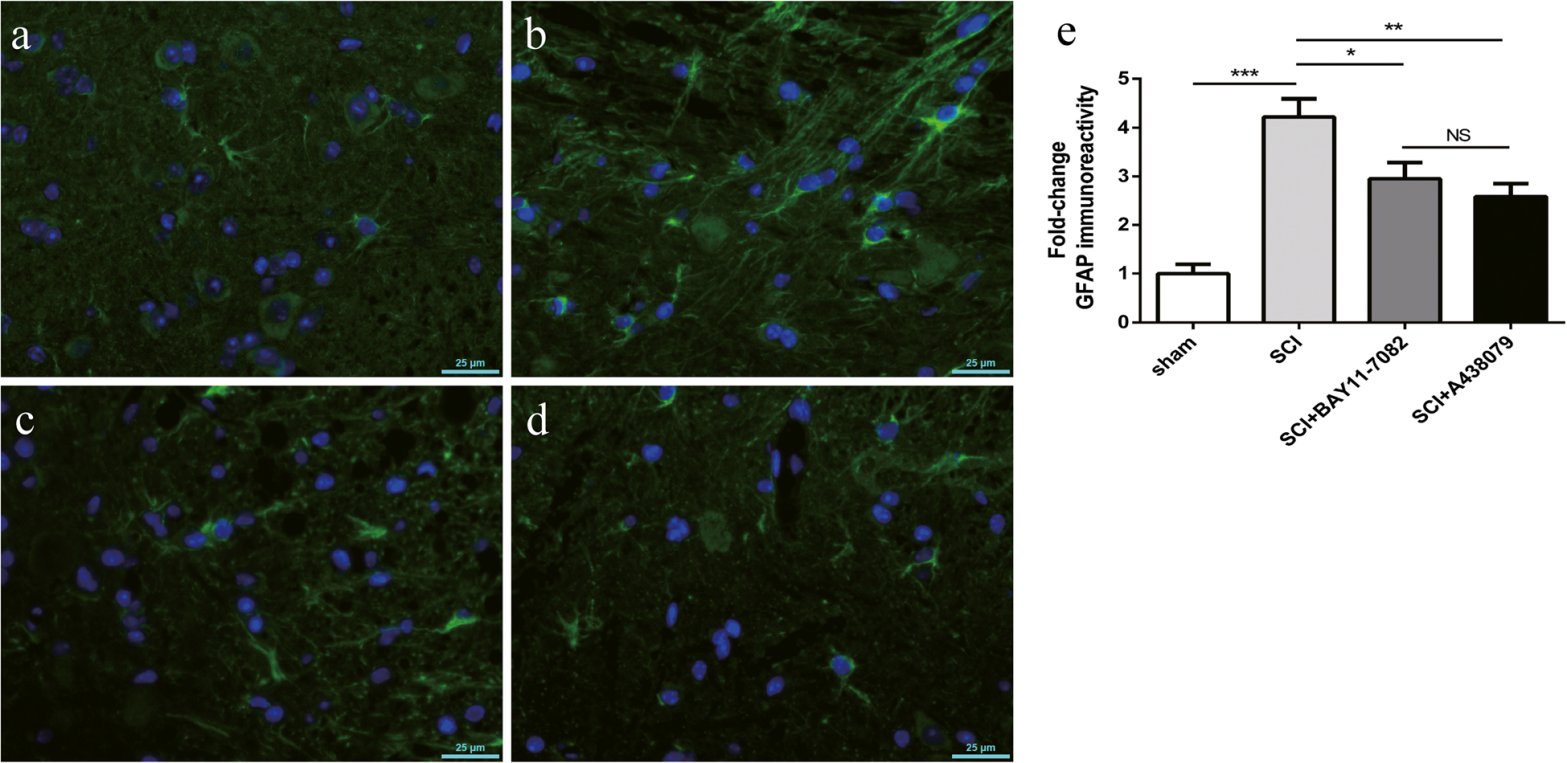
BAY 11-7082 or A438079 alleviates reactive gliosis. **a**–**d** Immunofluorescence labeling to estimate GFAP immunoreactivity (green fluorescence) 3 days after SCI. **a** SCI. **b** Sham. **c** SCI + BAY11-7082. **d** SCI + A438079. **e** Quantification analysis of GFAP immunoreactivity. HP high power (high-power field, 225 um × 162 um). *p < 0.05, **p < 0.01, ***p < 0.001, NS *p* > 0.05. Data are presented as the mean ± SEM, *n* = 5. Scale bars are 25 μm

To analyze the reactive astrogliosis that mediated the formation of glial scar [29], GFAP immunoreactivity was estimated by immunofluorescence labeling (Fig. 6a–d, green fluorescence) 3 days after SCI. GFAP immunoreactivity was robustly elevated after SCI (Fig. 6b, e, *P* < 0.001); these changes were markedly normalized by BAY 11-7082 (Fig. 6c, e, *P* < 0.05) or A438079 (Fig. 6d, e, *P* < 0.01). Furthermore, there was no significant difference between BAY 11-7082 and A438079 treatment (*P* > 0.05).

### Effects of NLRP3 inflammasome blockage on proinflammatory cytokines

**Fig. 7:**
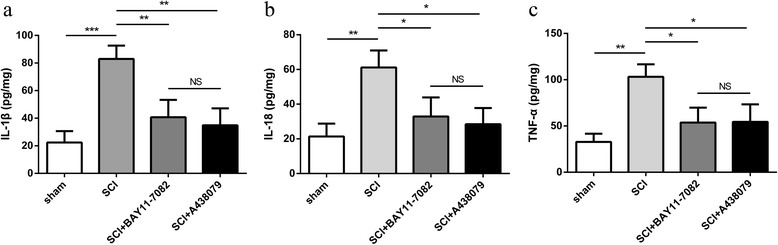
BAY 11-7082 or A438079 reduces proinflammatory cytokine levels. **a** Quantification analysis of interleukin (IL)-1β level in spinal cord 3 days after SCI. **b** Quantification analysis of IL-18 level in spinal cord 3 days after SCI. **c** Quantification analysis of tumor necrosis factor (TNF)-α level in spinal cord 3 days after SCI. *p < 0.05, **p < 0.01, ***p < 0.001, NS *p* > 0.05. Data are presented as the mean ± SEM, *n* = 5

The levels of proinflammatory cytokines, including IL-1β, IL-18, and TNF-α, were analyzed using ELISA based on previous studies [12, 13]. As shown in Fig. 7a–c, the protein levels of IL-1β (Fig. 7a, *P* < 0.001), IL-18 (Fig. 7b, *P* < 0.01), and TNF-α (Fig. 7c, *P* < 0.01) in the spinal cord tissue was significantly increased in the SCI group (*P* < 0.001). However, BAY 11-7082 or A438079 significantly attenuated these effects (Fig. 7a–c). Moreover, there was no significant difference between BAY 11-7082 and A438079 treatment (Fig. 7a–c, *P* > 0.05).

### Effects of NLRP3 inflammasome blockage on mitochondrial dysfunction

**Fig. 8:**
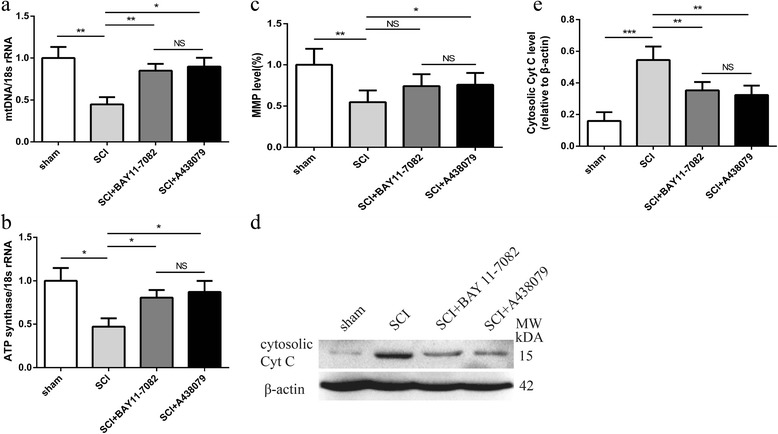
BAY 11-7082 or A438079 inhibits mitochondrial dysfunction. **a** Quantitative real-time PCR analysis of ATP synthase 3 days post injury. **b** Quantitative real-time PCR analysis of mitochondrial (mt) DNA 3 days post injury. **c** MMP level in the spinal cord tissue. **d** Representative western blots of cytosolic cytochrome c (Cyt C) 3 days post injury. **e** Quantification analysis of cytosolic Cyt C 3 days post injury. *p < 0.05, **p < 0.01, ***p < 0.001, NS *p* > 0.05. Data are presented as the mean ± SEM, *n* = 5

Based on previous study [10], we investigated the effects of BAY 11-7082 or A438079 on mitochondrial dysfunction through the changes of mtDNA copy number, ATP synthases, and the release of cytosolic Cyt C 3 days after SCI. SCI induced a significant abnormality of mitochondrial dysfunction, as demonstrated by reduction of mtDNA copy number (Fig. 8a, *P* < 0.01), ATP synthases (Fig. 8b, *P* < 0.05), MMP level (Fig. 8c, *P* < 0.01) and the release of cytosolic Cyt C (Fig. 8d, e, *P* < 0.001). Such abnormalities including the reduction of mtDNA copy number (BAY 11-7082: *P* < 0.01; A438079: *P* < 0.05), ATP synthases (BAY 11-7082: *P* < 0.05; A438079: *P* < 0.05) and the release of cytosolic Cyt C (BAY 11-7082: *P* < 0.01; A438079: *P* < 0.01) were strikingly reversed by BAY 11-7082 or A438079 (Fig. 8d–e).Furthermore, A438079 significantly attenuated the reduction of MMP level (Fig. 8c, *P* < 0.05). Moreover, no significant difference was found between BAY 11-7082 and A438079 treatment (Fig. 8a–e, *P* > 0.05).

### Effects of NLRP3 inflammasome blockage on NLRP3 inflammasome activation

**Fig. 9:**
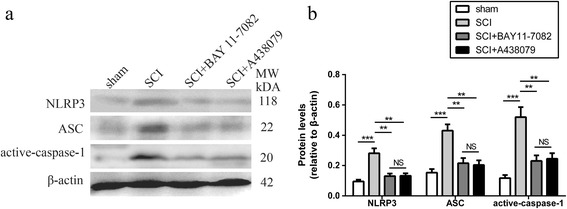
BAY 11-7082 or A438079 inhibits NLRP3 inflammasome activation. **a** Representative western blots of NLRP3, ASC, and active-caspase-1 3 days post injury. **b** Quantification analysis of NLRP3, ASC, and active-caspase-1 3 days post injury. **p < 0.01, ***p < 0.001, NS *p* > 0.05. Data are presented as the mean ± SEM, *n* = 5

NLRP3 inflammasome activation was analyzed through western blot. NLRP3 (Fig. 9a, b, *P* < 0.001), ASC (Fig. 9a, b, *P* < 0.001), and active-caspase-1 (Fig. 9a, b, *P* < 0.01) levels were increased in SCI mice 3 days post injury. Nevertheless, treatment with BAY 11-7082 or A438079 normalized these changes (Fig. 9a, b). Furthermore, no significant difference was found between BAY 11-7082 and A438079 treatment (*P* > 0.05). In addition, the pro-caspase-1 level did not significantly change in every group (data not shown).

## Discussion

Recently, it has been suggested that the NLRP3 inflammasome plays an important role in the molecular mechanism of diverse diseases [30, 31]. Inhibition of NLRP3 inflammasome activation provides protective effects in renal fibrosis [32] and inflammatory bowel disease [33]. Nevertheless, its role as pharmacologic inhibitors for drug therapy of SCI has been poorly investigated. Our results clearly demonstrate that inhibition of NLRP3 inflammasome activation by pharmacologic inhibitors controls neuroinflammation, alleviates mitochondrial dysfunction, and attenuates the severity of SCI in mice.

Efficacious NLRP3 inflammasome inhibitors are currently at an early stage of research and development. One widely used compound is BAY 11-7082 [19, 20, 34, 35], an I-κB kinase-β inhibitor, which selectively suppresses the ATPase activity of NLRP3 that is essential for its activation, and directly targets the NLRP3 inflammasome independently of inhibition of NF-κB activation [36, 37]. In our studies, this compound inhibited NLRP3 inflammasome activation, reduced spinal cord tissue damage, and improved functional recovery. Nevertheless, it is difficult to absolutely separate the role of inflammasome blockade from NF-kB blockade. In order to distinguish this point, another inflammasome blocking agent (A438079) [21, 38], which blocks the purinergic P2X7 receptor that is the upstream activator of the NLRP3 inflammasome [21, 38, 39], was used in this experiment. A438079 also resulted in suppression of NLRP3 inflammasome activation, promoted neuronal survival, and improved behavioral recovery in SCI mice.

Microglia/macrophage differentiation is divided into different phenotypes, with different subsets exerting distinct features [40, 41]. M1 phenotype microglia/macrophage are associated with high levels of pro-inflammatory cytokines, including TNF-α, and have detrimental effects and neurotoxicity [40]; whereas M2 phenotype microglia/macrophage produce anti-inflammatory mediators and exhibit homeostasis, regeneration, and neuroprotection [41]. Different subsets of microglia have been found in a variety of diseases [40, 41]. Additionally, it has been demonstrated that the balance of the M1/M2 microglia phenotype plays an important role in SCI [25, 41]. Promoting an M2 microglia shift alleviates damaging inflammatory activity and improves recovery processes [25, 41]. Here, we demonstrated that NLRP3 inflammasome blockade with BAY 11-7082 or A438079 decreased the number of CD68-positive cells, indicating the suppression of microglia/macrophage activation. Moreover, we analyzed microglia/macrophage polarization through investigation of the ratio of Arg1/iNOS mRNA based on previous study [16]. BAY 11-7082 or A438079 increased the ratio of Arg1/iNOS mRNA in our study. The results indicate that BAY 11-7082 or A438079 may promote microglia/macrophage phenotype to M2. However, further studies are required to clarify the effect of BAY 11-7082 or A438079 on microglia/macrophage polarization.

Neutrophils are believed to be crucial inflammatory cells in the spinal cord tissue after SCI [42].Moreover, neutrophils, secreting elastase, proteases, and MPO, result in demyelination, axonal degeneration, and the formation of lesions [42, 43].Inhibition of neutrophil accumulation improves sparing of white matter and promotes neurological recovery following SCI in mice [44]. In our study, we analyzed neutrophil infiltration using MPO activity detection based on previous study [28]. Our data showed that inhibition of NLRP3 inflammasome activation by BAY 11-7082 or A438079 attenuated MPO activity, suggesting that BAY 11-7082 or A438079 treatment suppresses neutrophils invasion.

Reactive astrocytes contribute to glial scar formation after SCI [29]. Glial scar results in a physical and biochemical barrier of axon regeneration, and thus affects neurologic functional recovery after SCI [45, 46]. Attenuation of reactive astrocytes promoted function recovery after SCI [29]. We found that inhibition of NLRP3 inflammasome activation by BAY 11-7082 or A438079 significantly attenuated GFAP immunoreactivity, suggesting that reactive astrocytes are inhibited by BAY 11-7082 or A438079.

Mitochondria are important intracellular organelles in controlling cellular energy metabolism [47]. Moreover, mitochondria are a major source of reactive oxygen species (ROS) and contribute to cell signal transduction [10, 48]. Mitochondrial dysfunction leads to the increase of ROS production, mitochondrial DNA damage (copy number reduction and mutation), disorders of oxidative phosphorylation of the mitochondrial respiratory chain, and the reduction of ATP production [10, 48]. Mitochondrial dysfunction plays important role in neuronal cell death [49]. Regulation of mitochondrial dysfunction promotes neurological recovery after SCI [5, 49]. In the present study, we analyzed mitochondrial dysfunction using the changes of mtDNA copy number, ATP synthases, and the release of cytosolic Cyt C based on previous studies [10, 48]. Our results showed that inhibition of NLRP3 inflammasome activation by BAY 11-7082 or A438079 reversed the reduction of mtDNA copy number and ATP synthases and the increase of cytosolic Cyt C. Moreover, A438079 attenuated the reduction of MMP level. These results indicate that BAY 11-7082 or A438079 may attenuate mitochondrial dysfunction following SCI in mice.

NLRP3 inflammasome is activated after SCI [12, 13, 50–52], and NLRP3 inflammasome expression is significantly increased 3 days post SCI in rats [12, 13, 51, 52]. Moreover, NLRP3 is expressed primarily in neurons and to a lesser extent in microglia and astroglia [13]. The neuroprotection of dopamine D1 receptor agonist [12], stromal cell-derived factor-1 alpha [13], d-β-hydroxybutyrate [15], wogonoside [16], quercetin [51], and asiatic acid [52] against SCI presumably depend on the inhibition of NLRP3 inflammasome activation to suppress neuroinflammation. Here, we showed that the mRNA expression of NLRP3 immediately increased within the first 6 h, further rose until at day 3 and dropped at day 7 post injury. Furthermore, protein expressions of NLRP3 and active-caspase-1 significantly increased 72 h after SCI in mice, which is consistent with previous study [13]. Moreover, protein levels of NLRP3, ASC, active-caspase-1, and proinflammatory cytokines, including IL-1β and IL-18, was reduced by pharmacologic inhibitors BAY 11-7082 or A438079, suggesting that NLRP3 inflammasome activation is inhibited by BAY 11-7082 or A438079. BAY 11-7082 or A438079 may thus have an important therapeutic profile, capably controlling the earliest steps in inflammatory cascades.

## Conclusions

Collectively, our results demonstrate that pharmacologic suppression of NLRP3 inflammasome activation controls neuroinflammation, attenuates mitochondrial dysfunction, alleviates the severity of spinal cord damage, and improves neurological recovery after SCI. These data strongly indicate that the NLRP3 inflammasome is a vital contributor to secondary damage after SCI in mice.

## Abbreviations

ANOVA: Analysis of variance
Arg1: Arginase1
ASC: Apoptosis-associated speck-like protein containing a caspase recruitment domain
BMS: Basso Mouse Scale
Cyt C: Cytosolic cytochrome c
GAPDH: Glyceraldehyde-3- phosphate dehydrogenase
GFAP: Glial fibrillary acidic protein
IL: Interleukin
iNOS: Inducible nitric oxide synthase
MMP: Mitochondrial membrane potential
MPO: Myeloperoxidase
mt: Mitochondrial
NLRP3: NOD-like receptor protein-3
qPCR: Quantitative real-time polymerase chain reaction
SCI: Spinal cord injury
SEM: Standard error of the mean
ROS: Reactive oxygen species
TNF: Tumor necrosis factor
TUNEL: Terminal dexynucleotidyl transferase-mediated dUTP nick end labeling
